# Non-invasive vagus nerve stimulation in anti-inflammatory therapy: mechanistic insights and future perspectives

**DOI:** 10.3389/fnins.2024.1490300

**Published:** 2024-11-13

**Authors:** Fu-Jun Liu, Jing Wu, Li-Jun Gong, Hong-Shuai Yang, Huan Chen

**Affiliations:** ^1^Neurology Medical Center II, Foresea Life Insurance Guangzhou General Hospital, Guangzhou, China; ^2^Department of Medical Imaging, Foresea Life Insurance Guangzhou General Hospital, Guangzhou, China; ^3^Center of Surgical Anesthesia, The Sixth Affiliated Hospital of Sun Yat-sen University, Guangzhou, China; ^4^Central Operating Room, Foresea Life Insurance Guangzhou General Hospital, Guangzhou, China; ^5^Department of Cell Biology, School of Basic Medical Sciences, Southern Medical University, Guangzhou, China

**Keywords:** vagus nerve stimulation (VNS), anti-inflammatory therapy, cholinergic anti-inflammatory pathway (CAP), neuro-immune reflex, clinical applications, precision neuromodulation, non-invasive therapy, chronic inflammation

## Abstract

Non-invasive vagus nerve stimulation (VNS) represents a transformative approach for managing a broad spectrum of inflammatory and autoimmune conditions, including rheumatoid arthritis and inflammatory bowel disease. This comprehensive review delineates the mechanisms underlying VNS, emphasizing the cholinergic anti-inflammatory pathway, and explores interactions within the neuro-immune and vagus-gut axes based on both clinical outcomes and pre-clinical models. Clinical applications have confirmed the efficacy of VNS in managing specific autoimmune diseases, such as rheumatoid arthritis, and chronic inflammatory conditions like inflammatory bowel disease, showcasing the variability in stimulation parameters and patient responses. Concurrently, pre-clinical studies have provided insights into the potential of VNS in modulating cardiovascular and broader inflammatory responses, paving the way for its translational application in clinical settings. Innovations in non-invasive VNS technology and precision neuromodulation are enhancing its therapeutic potential, making it a viable option for patients who are unresponsive to conventional treatments. Nonetheless, the widespread adoption of this promising therapy is impeded by regulatory challenges, patient compliance issues, and the need for extensive studies on long-term efficacy and safety. Future research directions will focus on refining VNS technology, optimizing treatment parameters, and exploring synergistic effects with other therapeutic modalities, which could revolutionize the management of chronic inflammatory and autoimmune disorders.

## 1 Introduction

Inflammation is a vital biological response that protects the body from harmful stimuli, such as pathogens, damaged cells, and irritants (Netea et al., 2017). This complex process, marked by the coordinated activation of immune and non-immune cells, is essential for pathogen clearance and tissue repair. However, inflammation can also be a double-edged sword. Acute inflammation is a rapid, self-limiting response critical for immediate defense and healing (Hannoodee and Nasuruddin, 2020). In contrast, chronic inflammation is a prolonged, dysregulated process that can persist for months or even years, often without an overt pathogen. Chronic inflammation is a key driver of numerous diseases, including autoimmune disorders, cardiovascular diseases, metabolic syndrome, neurodegenerative conditions, and various cancers (Furman et al., 2019; Christ et al., 2019; Dugan et al., 2023). Collectively, these inflammation-related conditions pose a significant global health burden, accounting for more than half of all deaths worldwide (Yu et al., 2024).

Despite the availability of various anti-inflammatory therapies, managing chronic inflammation effectively remains a formidable challenge (Blagov et al., 2024; Feehan and Gilroy, 2019). Current drug therapy, especially non-steroidal anti-inflammatory drugs (NSAIDs), can cause serious gastrointestinal complications such as peptic ulcers and gastritis, especially with long-term use. In addition to impairing organ function, they also increase the risk of cardiovascular disease, including heart attack and stroke. Biologic therapies, while targeted and effective against specific inflammatory pathways, also carry the risk of immunosuppression. This can make patients more susceptible to infections and, in some cases, lead to the development of malignancies. In addition, biologics may induce autoantibodies and, in rare cases, autoimmune diseases. The use of certain anti-inflammatory drugs also carries the risk of hepatotoxicity and renal dysfunction, and therefore requires regular monitoring and caution when used in patients with pre-existing liver or kidney disease. The urgent need for novel, targeted approaches that can modulate the immune response with greater precision and safety. In this context, VNS has emerged as a promising therapeutic strategy by leveraging the body's intrinsic neuroimmune pathways to control inflammation (Browning et al., 2017; Chen et al., 2023). Originally developed for neurological conditions such as epilepsy and depression, VNS has been repurposed to harness its anti-inflammatory potential (Bazoukis et al., 2023). By engaging multiple mechanisms—including the cholinergic anti-inflammatory pathway (Song et al., 2024; Bonaz et al., 2016b), the vagus-adrenal axis, and the vagus-gut axis—VNS offers a level of precision in modulating systemic inflammation that traditional therapies cannot achieve.

The clinical applications of VNS are rapidly expanding beyond its established role in treating rheumatoid arthritis and inflammatory bowel disease (Hays et al., 2023). Emerging evidence supports its efficacy across a wide range of inflammatory conditions, including sepsis, cardiovascular diseases, autoimmune disorders, and chronic pain syndromes (Zafeiropoulos et al., 2024; Dawson et al., 2021; Tao et al., 2020; Shao et al., 2023; Courties et al., 2021; Ridengnaxi and Wang, 2024). Innovations in VNS technology, such as non-invasive devices and personalized stimulation protocols, are further enhancing its therapeutic potential and accessibility. This review presents a comprehensive examination of the mechanistic pathways engaged by VNS, its evolving clinical applications from traditional to emerging inflammatory conditions, and the latest technological innovations that enhance its therapeutic potential. By integrating clinical outcomes with mechanistic insights from pre-clinical models, our review offers a unique synthesis aimed at advancing the integration of VNS into mainstream anti-inflammatory therapy.

## 2 Mechanisms of vagus nerve stimulation in anti-inflammatory therapy

Recent advancements have expanded the clinical applications of vagus nerve stimulation (VNS) in treating inflammatory diseases. Table 1 summarizes key pre-clinical studies demonstrating the anti-inflammatory effects of VNS in various animal models. These foundational studies have paved the way for exploring VNS in clinical settings. Additionally, the clinical efficacy of VNS across different inflammatory diseases is outlined in Table 2, highlighting its therapeutic potential in conditions such as rheumatoid arthritis, inflammatory bowel disease, and others.

**Table 1:** Pre-clinical studies on VNS in animal models of inflammation.

**References**	**Animal species**	**Disease model**	**Stimulation parameters**	**Primary outcomes**	**Key findings**
Caravaca et al. (2022a)	Mice	Zymosan-induced peritonitis	1 mA, 250-μs biphasic pulse, 10 Hz, 5 min	Reduction in inflammation duration, Increased efferocytosis	VNS promoted resolution of inflammation, higher levels of SPMs, dependent on Alox15 and α7nAChR
Meregnani et al. (2011)	Rats	TNBS-induced colitis	1 mA, 5 Hz, 500 μs pulse width, 3 h/day for 5 days	Reduced body weight loss, decreased MPO levels, lower histological inflammation scores	Chronic VNS significantly mitigated inflammation in colitis, with effects on cytokine mRNA levels and MPO activity
Meroni et al. (2018)	BALB/c mice	Oxazolone-induced colitis	Electrical VNS: 1 mA, 1 ms, 5 Hz, 5 min	Improved survival rates, reduced systemic inflammation	VNS significantly improved survival, reduced serum HMGB1 levels, and attenuated systemic inflammatory responses in a Th2-driven colitis model.
Olofsson et al. (2012)	Mice	Inflammatory stimulation	Vagus nerve stimulation, 1 mA, 10 Hz, 1 min	Inhibition of TNF production	α7nAChR expression on bone marrow-derived non-T cells essential for vagus nerve-mediated TNF suppression; not required on neurons or T cells.
Chen et al. (2018)	Mice	Cerebral Microinfarct with Colitis	0.5 mA, 5 Hz, 30 s every 5 min for 1 h, performed during acute colitis phase	Reduced microinfarct volume, improved BBB integrity	VNS significantly reduced neuroinflammation and BBB permeability, mitigating CMI and colitis-induced CMI aggravation.
Caravaca et al. (2022b)	Rats	Indomethacin-induced enteropathy	1 mA, 200 μs pulse width, 50 μs inter-pulse-interval, 60 s at 10 Hz	Reduced intestinal lesion area, decreased systemic and intestinal pro-inflammatory cytokines	VNS effectively reduced small bowel inflammation independent of the spleen, highlighting a potential therapeutic pathway for IBD.
Namgung et al. (2022)	Rats	Continuous stress model	Acute VNS: 10 mA, 5 Hz, 5 ms pulse duration, 5 min	Reduction in inflammatory cytokines in hippocampal and gastric tissues	VNS modulated hippocampal inflammation by altering microglial activation and cytokine production.
Li et al. (2021)	Rats	LPS-induced ARDS	Electrical VNS: 5 V, 5 Hz, 2 ms, 10 min post-LPS	Reduced lung injury, modulation of macrophage phenotypes, decreased pyroptosis	VNS reduces lung inflammation in ARDS through modulation of macrophage phenotypes and inhibition of pyroptosis, largely dependent on α7nAchR and STAT3 activation.
Payne et al. (2019)	Sprague-Dawley rats	TNBS-induced IBD	Sub-diaphragmatic VNS, 320 nC per phase, 10 Hz, 3 h/day	Improved stool quality, no blood in feces, reduced CRP	Abdominal VNS reduced inflammation effectively with fewer off-target effects compared to cervical VNS.
Huang et al. (2024)	Mice	LPS-induced ALI	50 μA, 5 Hz, 1 ms, 20 min	Decreased inflammatory cytokines, reduced lung injury	VNS modulated α7nAChR which increased Netrin-1 expression, resulting in increased synthesis of LXA4 and improved lung injury outcomes.
Zhang S. J. et al. (2021)	Beagles	Rapid atrial pacing (RAP)-induced atrial fibrillation (AF)	Low-level VNS: 2 Hz, 0.2 ms pulse duration, 80% of threshold voltage for 3 h	Increased effective refractory period (ERP), reduced AF inducibility	LL-VNS modulated inflammatory cytokine levels (TNF-α, IL-6), increased STAT3 expression, and reduced NF-κB expression, demonstrating that LL-VNS modulates AF vulnerability through the α7nAChR-mediated cholinergic anti-inflammatory pathway (CAP).
Jin et al. (2017)	Rats	TNBS-induced colitis	VNS: 25 Hz, 2 s on/3 s off, 0.5 ms pulse width, 1.0–3.0 mA, 3 h/day for 21 days	Decreased DAI, improved CMDI and histological scores	Chronic VNS reduced pro-inflammatory cytokines (TNF-α, IL-1β, and IL-6) and enhanced vagal activity. Addition of EA further improved DAI, CMDI, and histological scores, demonstrating a synergistic effect.
Xie et al. (2023)	Mice	tMCAO-induced ischemic stroke	Invasive VNS: 0.5 mA, 5 Hz, 30 s every 5 min for 1 h, repeated at 30 min, 24 h, and 48 h after tMCAO	Reduced cerebral infarct volume, improved neurological function	VNS attenuated neuroinflammation and reduced neurological deficits by upregulating USP10, which inhibited the NF-κB signaling pathway. USP10 silencing reversed the protective effects of VNS.
Hosomoto et al. (2023)	Rats	6-OHDA-induced Parkinson's disease	Continuous VNS: 30 Hz, 0.1 mA (days 0–7), 0.25 mA (days 7–14), 500 μs pulse width, 30 s on/5 min off	Reduced motor impairments, decreased glial cell activation, increased LC-DbH density	Continuous VNS and afferent VNS reduced motor deficits, decreased microglial activation in the SNpc, and increased TH-positive neurons, highlighting the role of afferent pathways.
Zhao et al. (2013)	Rats	Myocardial ischemia/reperfusion (I/R) injury	Electrical VNS: 5 Hz, 1 ms pulse duration, 2–6 V for 75 min	Reduced infarct size, decreased TNF-α and IL-1β levels, improved endothelial function	VNS protected against myocardial I/R injury by activating the cholinergic anti-inflammatory pathway through α7nAChR, reducing systemic and vascular inflammatory cytokine levels, and modulating the STAT3 and NF-κB signaling pathways.
Levine et al. (2014)	Rats	Collagen-induced arthritis (CIA)	VNS: 3 mA, 10 Hz, 200 μs pulse width, 60 s daily for 7 days	Reduced ankle swelling, decreased histological arthritis score	VNS reduced arthritis severity by activating the cholinergic anti-inflammatory pathway, resulting in decreased joint inflammation and bone erosion.
Wang et al. (2012)	Rats	Myocardial ischemia/reperfusion (I/R) injury	Electrical VNS: 10 Hz, 2 ms pulse width, adjusted to 10% HR reduction for 30 min post-conditioning	Decreased infarct size, reduced serum levels of TNF-α, IL-1, IL-6, HMGB-1, and ICAM-1	VNS post-conditioning significantly reduced local and systemic inflammatory responses and provided cardioprotection against I/R injury through activation of the cholinergic anti-inflammatory pathway.
Sun et al. (2013)	Rats	TNBS-induced colitis	Chronic VNS: 0.25 mA, 20 Hz, 500 μs pulse width, 30 s ON/5 min OFF for 3 h/day	Reduced disease activity index, improved CMDI scores, decreased MPO, iNOS, TNF-α, and IL-6 levels	Chronic VNS reduced colonic inflammation via modulation of the MAPK/NF-κB signaling pathway and enhanced vagal activity, indicating the activation of the cholinergic anti-inflammatory pathway.
Zhang L. et al. (2021)	Mice	Caerulein-induced acute pancreatitis, Pancreatic duct ligation (PDL)	Electrical VNS: 0.3 mA, 10 Hz, 2 min (single session)	Reduced pancreatic amylase and lipase levels, decreased histological damage in pancreas, improved survival in PDL	VNS reduced systemic inflammation and pancreatic damage in both mild (Caerulein) and severe (PDL) AP models. The protective effects were mediated through the upregulation of α7nAChR+ macrophages, independent of spleen involvement.
Kin et al. (2021)	Rats	6-OHDA-induced Parkinson's Disease	VNS: 0.1, 0.25, 0.5, and 1 mA, 30 Hz, 500 μs pulse width, 30 s ON/5 min OFF for 14 days	Improved motor behavior, decreased microglia and astrocyte activation, increased DA neuron survival	VNS at 0.25–0.5 mA showed maximal anti-inflammatory and neuroprotective effects, preserving both DA and NA neurons. Lower (0.1 mA) and higher (1 mA) intensities were less effective.
Mihaylova et al. (2012)	Sprague-Dawley Rats	LPS-induced endotoxemia	VNS: 2 mA, 0.3 ms pulse width, 2 Hz, 10 min every 45 min	Stabilized cerebral microcirculation, reduced inflammatory cytokines (IL-6, TNF-α)	VNS mitigated early sepsis-associated neurovascular dysfunction, stabilized cerebral blood flow, and improved neurovascular coupling by reducing systemic and cerebral inflammation.
Jo et al. (2020)	Rats	Concanavalin A (ConA)-induced hepatitis	Hepatic VNS: 10 mA, 5 Hz, 5 ms pulse duration, 5 min, single session	Reduced TNF-α, IL-1β, and IL-6 mRNA and protein levels, decreased ALT and AST	VNS activated vagal afferent fibers and regulated hepatic cytokine production via α7 nAChR and STAT3 signaling pathways, reducing liver inflammation and immune cell recruitment
Guo et al. (2019)	Rats	TNBS-induced colitis, DSS-induced colitis	Sacral nerve stimulation (SNS): 2 mA, 0.3 ms pulse width, 5 Hz, 10 s ON/90 s OFF, 3 h/day for 10 days	Reduced disease activity index (DAI), normalized colon length, decreased spleen weight, reduced MPO activity	SNS significantly reduced colonic inflammation, similar to the effects of vagus nerve stimulation (VNS), by modulating autonomic function and cytokine production (decreased TNF-α, IL-6, and IL-17A, increased IL-10), indicating potential therapeutic utility for IBD treatment.

**Table 2:** Clinical applications of vagus nerve stimulation (VNS) in inflammatory diseases.

**References**	**Patient demographics**	**Disease/condition**	**Stimulation parameters**	**Outcome measures**	**Key findings**
Koopman et al. (2016)	RA patients, seven female, 11 male, ages 25–43	Rheumatoid arthritis	0.25–2.0 mA; 250-μs pulse width; 10 Hz; 60 s stimulation up to four times daily	Significant reduction in TNF production and disease severity improvement	VNS inhibits TNF production and reduces inflammation, improving clinical signs and symptoms in RA patients
Bonaz et al. (2016a)	Seven patients (four male, three female), ages 20–51, two smokers, five non-smokers	Crohn's disease (CD)	0.25–1.25 mA; 30 s ON/5 min OFF; 500 μs pulse width; 10 Hz	Clinical, biological, and endoscopic remission; Restored vagal tone	VNS well-tolerated, effective in inducing remission in active CD over 6 months. Increased vagal tone correlated with disease improvement
Sahn et al. (2023)	22 patients (10 CD, 12 UC), Ages 10–21, 55% male	Pediatric-onset Crohn's Disease (CD) and Ulcerative Colitis (UC)	Transcutaneous auricular VNS, 20 Hz, 300 μs, 5 min duration once daily for 2 weeks, then twice daily until week 16	Clinical remission, ≥50% reduction in fecal calprotectin from baseline to week 16	ta-VNS attenuated signs and symptoms in pediatric IBD, significantly reducing fecal calprotectin levels with good safety and tolerability
Koopman et al. (2016)	18 patients, mixed sex, ages 36–69	Rheumatoid arthritis (RA)	0.25–2.0 mA; 250-μs pulse width; 10 Hz; up to four times daily	Reduced TNF production, improved clinical scores (DAS28-CRP)	VNS significantly reduced TNF levels and improved RA symptoms, showing potential as a treatment for autoimmune diseases
Baumgart et al. (2016)	212 patients (CD: 97, UC: 115), mixed gender, ages 10–69	Crohn's disease (CD) and Ulcerative colitis (UC)	Vedolizumab 300 mg IV at week 0, 2, 6, then every 8 weeks	Clinical remission at week 14, steroid-free clinical remission, clinical response	Vedolizumab was effective in routine clinical use, especially for patients who were anti-TNFα naive. Reductions in CRP and calprotectin noted, indicating decreased inflammation
D'Haens et al. (2023)	17 patients, 76.5% male, ages 21–62, predominantly Caucasian	Crohn's disease (CD)	Implanted device, 0.25–2.0 mA, 250 μs, 10 Hz, 1–5 min once to four times daily	CDAI reduction, fecal calprotectin decrease, quality of life improvement	VNS significantly reduced CD activity and inflammation with improvement in quality of life over 16 weeks
Sinniger et al. (2020)	Nine patients (five men, four women), Median age: 39 years, Range: 20–52 years	Crohn's disease	Left cervical VNS, 0.25 mA to 1.5 mA, 250–500 μs, 10 Hz, 30 s ON/5 min OFF, continuous for 1 year	Clinical and endoscopic remission, changes in CRP and fecal calprotectin, HRV	VNS showed significant reduction in inflammation and improvement in clinical and endoscopic outcomes in CD, with changes in cytokine profiles and HRV indicating systemic and gut-specific anti-inflammatory effects
Venborg et al. (2021)	15 patients, predominantly Caucasian, 87% female, Mean age: 65 years	Polymyalgia rheumatica	t-VNS, 2 min stimulation three times daily for 5 days	Cardiac vagal tone (CVT), heart rate, pain VAS score, CRP	t-VNS increased CVT and decreased heart rate; pain related to the hips decreased by 14%; no significant change in CRP or other pro-inflammatory markers
Kaur et al. (2023)	Pediatric patients, ages 5–18 years, mixed gender	Medically refractory epilepsy	VNS settings: 1.75–2.25 mA, 20–30 Hz, 250–500 μs pulse width, 30 s ON, 1.8–5 min OFF	Gene expression changes in PBMCs, inflammatory markers	VNS demonstrated anti-inflammatory effects by downregulating genes associated with stress, inflammation, and immune responses in pediatric epilepsy patients
Aranow et al. (2021)	18 patients (16 women, 2 men), Mean age: 48 years, Racial distribution: 58% White, 35% African American	Systemic Lupus Erythematosus (SLE)	taVNS: 30 Hz, 300 μs, 5 min daily for 4 days, left ear concha	Reduced pain (VAS), reduced fatigue (FACIT-F), decreased tender and swollen joint counts	taVNS significantly reduced pain and fatigue compared to sham, with greater reductions in plasma substance P levels, suggesting anti-inflammatory and analgesic effects of taVNS
Stavrakis et al. (2024)	26 female patients, Mean age: 34 years, 81% Caucasian	Postural Tachycardia Syndrome (POTS)	tVNS: 20 Hz, 1 mA, tragus stimulation, 1 h daily for 2 months	Reduction in orthostatic tachycardia, decreased inflammatory cytokines (TNF-α), improved heart rate variability	tVNS significantly attenuated orthostatic tachycardia, reduced antiadrenergic autoantibodies, and improved autonomic tone with no device-related side effects.
Stavrakis et al. (2017)	54 patients (26 in LLVNS group, 28 in control group), Mean age: 57.5 years, 70% male	Post-Operative Atrial Fibrillation (POAF)	LLVNS: 20 Hz, 0.1 ms pulse width, continuous stimulation for up to 72 h, set at 50% of bradycardia threshold	Incidence of POAF, serum TNF-α, IL-6, IL-10, CRP levels, gene expression	LLVNS significantly reduced POAF incidence (12 vs. 36%, *p* = 0.027) and decreased serum levels of TNF-α and IL-6 compared to controls. No stimulation-related complications observed.
Okdahl et al. (2024)	131 patients (68 Type 2, 63 Type 1), Mean age: 56.5 years, 59% female	Type 1 and Type 2 Diabetes with Autonomic Neuropathy	tVNS: 25 Hz, 5 kHz sine waves, 120 s per session, four times daily for 7 days (high-dose), then twice daily for 56 days (moderate-dose)	Inflammatory cytokines (IL-6, IL-8, IL-10, TNF-α, and IFN-γ), cardiac autonomic neuropathy (CAN), HRV	tVNS did not show any significant reduction in inflammatory cytokines compared to sham, regardless of diabetes type, autonomic function status, or stimulation intensity. No serious adverse events reported.
Stavrakis et al. (2015)	40 patients (20 in LLTS group, 20 in control group), Mean age: 61.9 years, 70% male	Paroxysmal Atrial Fibrillation (AF)	LLTS: 20 Hz, 1 ms pulse width, set at 50% of the voltage slowing sinus rate; applied for 1 h during AF induction	AF inducibility, AF duration, AF cycle length, systemic TNF-α, IL-6, and CRP levels	LLTS significantly reduced AF duration (10.4 vs. 18.5 min in control), prolonged AF cycle length, and decreased systemic TNF-α and CRP levels, indicating antiarrhythmic and anti-inflammatory effects.
Wu et al. (2023)	20 patients with sepsis, Mean age: 68 years, 75% male	Sepsis	taVNS: 25 Hz, 3 mA, 500 μs, 30 min daily for 5 days	Changes in serum cytokines (TNF-α, IL-1β, IL-6, IL-4, and IL-10), APACHE II and SOFA scores	taVNS significantly reduced pro-inflammatory cytokines (TNF-α, IL-1β) and increased anti-inflammatory cytokines (IL-4, IL-10). SOFA scores decreased by day 5, but no significant changes were found in APACHE II scores. No adverse effects observed
Bellocchi et al. (2023)	32 SSc patients; 21 completed study; 90% female; Mean age: 58 years, 86% Caucasian	Systemic Sclerosis (SSc)	tVNS: 25 Hz, 250 μs, 30 s ON/30 s OFF, 4 h/day for 4 weeks	Pain reduction (Numeric Rating Scale), IL-6, HRV, PROMIS-29 Item 4, PHQ-9, PSQI	tVNS significantly reduced pain (−27.7 vs. −7.7% in control, *p* = 0.002) and IL-6 levels. No significant changes in HRV or QoL indices. Safe, with no adverse effects.
Sinniger et al. (2020)	Nine patients (five male, four female); Median age: 39 years; Range: 20–52 years	Crohn's Disease (CD)	Implanted device: 30 s ON/5 min OFF, 10 Hz, 0.25–1.25 mA, continuous for 12 months	CDAI, CDEIS, CRP, fecal calprotectin, cytokine profiles, HRV	VNS was well-tolerated, with 5 patients in clinical remission and 6 in endoscopic remission at 12 months. Significant reductions in inflammatory markers (CRP, fecal calprotectin, IL-6, IL-23, and TNF-α) and restored vagal tone
Kox et al. (2015)	20 healthy male volunteers, mean age: 24 years (sham), 26 years (tVNS)	Experimental Human Endotoxemia	tVNS: 2–10 V, 1 ms pulse width, 20 Hz, continuous for 30 min starting 10 min before LPS administration	Plasma cytokines (TNF-α, IL-6, IL-8, and IL-10), neutrophil phagocytosis, leukocyte counts, hemodynamic parameters	tVNS was feasible and safe but did not modulate plasma cytokine levels, neutrophil function, or hemodynamic responses during endotoxemia, indicating no significant anti-inflammatory effect in this human model

### 2.1 Cholinergic anti-inflammatory pathway and neuro-immune reflexes

#### 2.1.1 Cholinergic anti-inflammatory pathway

The cholinergic anti-inflammatory pathway (CAP) is central to the mechanism by which VNS modulates inflammation, primarily through acetylcholine (ACh) interactions with α7 nicotinic acetylcholine receptors (α7nAChR) on macrophages. This targeted interaction inhibits the release of pro-inflammatory cytokines such as Tumor necrosis factor alpha (TNF-α), Interleukin-1beta (IL-1β), and High Mobility Group Box 1 (HMGB1), effectively curtailing the inflammatory response, thus offering a more focused approach than systemic immunosuppressive therapies, which often result in broader immune suppression (Han et al., 2017; Kelly et al., 2022; Noviello et al., 2021). Introduced by Tracey in 2002, CAP has been extensively validated, showing its efficacy in reducing neuroinflammation and its beneficial impact on chronic conditions like autoimmune diseases and metabolic syndrome, with fewer associated side effects such as reduced risk of systemic infections, malignancy development, and organ toxicity typically seen with broader immunosuppressive treatments (Tracey, 2002; Qin et al., 2020; Lv et al., 2021). Additionally, CAP's interaction with biological pathways including those involving the gut microbiota and endocrine systems highlights its extensive regulatory influence on metabolic balance and energy homeostasis (Hilderman and Bruchfeld, 2020).

Moreover, while the direct modulation of inflammation via vagal nerve stimulation by CAP is well-established, recent research highlights a more complex mechanism involving the peripheral nervous system and immune cells. Specifically, the inflammatory reflex mediated by the vagus nerve critically regulates cytokine production, notably TNF-α, through acetylcholine signaling in spleen macrophages (Tracey, 2002). However, this pathway is not exclusively cholinergic; it also involves the activation of the sympathetic nervous system and β_2_-adrenergic receptors. VNS has been shown to activate splenic sympathetic nerve fibers, leading to the release of norepinephrine, which interacts with β_2_-adrenergic receptors on acetylcholine-producing T cells, thereby promoting their release of acetylcholine (Rosas-Ballina et al., 2011; Inoue et al., 2019). This circuitry is bolstered by the discovery of acetylcholine-producing, memory phenotype T cells in the spleen, which are pivotal due to the absence of enzymatic machinery for acetylcholine synthesis in spleen nerve fibers. Thus, the interplay between the parasympathetic and sympathetic nervous systems is crucial for modulating immune function via the cholinergic anti-inflammatory pathway. These T cells, activated by vagus nerve-induced action potentials, secrete acetylcholine to effectively control innate immune responses. This demonstrates a dynamic interplay between the nervous and immune systems, furthering our understanding of CAP's role in anti-inflammatory therapy. The advanced insights provided by these studies illustrate how neurotransmitters like acetylcholine are critical mediators between neural and immune regulation, expanding the potential therapeutic applications of CAP in reducing inflammatory diseases without the broader side effects associated with systemic immunosuppressive therapies.

#### 2.1.2 Reflexive anti-inflammatory mechanisms

The Neuro-Immune Reflex represents a critical communication pathway between the nervous and immune systems, mediated through both afferent (sensory) and efferent (motor) branches. Central to the rapid modulation of inflammation through VNS are reflexive anti-inflammatory pathways (Pavlov and Tracey, 2012). The inflammatory reflex, initiated by afferent vagal signals from peripheral inflammation, is processed by the nucleus tractus solitarius (NTS) in the brainstem (Liu et al., 2024). The NTS then orchestrates efferent vagal outputs that inhibit pro-inflammatory cytokines, particularly TNF-α, via the cholinergic anti-inflammatory pathway (CAP). The combination of the afferent and efferent arms of this vagal-immune interaction is termed the “inflammatory reflex” (Kelly et al., 2022), this reflex enables quick and localized suppression of inflammation, essential in acute conditions like sepsis.VNS significantly reduces TNF levels in murine endotoxemia, an effect lost in animals with a severed vagus nerve (Borovikova et al., 2000). Additionally, VNS affects cardiovascular reflexes (Pavlov, 2021), such as the baroreflex, which regulates heart rate and vascular tone, thus indirectly moderating systemic inflammation. The interaction between these reflexive mechanisms enhances VNS's effectiveness in addressing both inflammatory and cardiovascular disturbances, providing a multifaceted approach to managing acute inflammatory responses.

#### 2.1.3 Spleen-mediated immune modulation

Research indicates that the efferent arm of the inflammatory reflex is likely the sympathetic nervous system, not the CAP, as action potentials are transmitted through the sympathetic chain and splanchnic nerves to the splenic nerve, inhibiting cytokine release (Martelli et al., 2014). Although the spleen is not directly innervated by the vagus nerve, it is integral to its anti-inflammatory impact, with mediation occurring through splanchnic nerves and T cells (Rosas-Ballina et al., 2011, 2008). Recognized increasingly for its role in inflammation modulation, the spleen is stimulated by VNS via the splenic nerve. This stimulation leads to the release of acetylcholine, which binds to α7 nicotinic acetylcholine receptors (α7nAChRs) on splenic macrophages. This binding significantly curtails the release of pro-inflammatory cytokines like TNF-α, thereby diminishing systemic inflammation (Falvey et al., 2022; Wang et al., 2021). This pathway is particularly vital in chronic inflammatory diseases where persistent cytokine production drives disease activity. VNS offers a novel, non-invasive approach to managing conditions such as rheumatoid arthritis and lupus, providing a safer, more precise alternative to traditional immunosuppressive therapies and underscoring its potential for targeted immune modulation through the spleen.

#### 2.1.4 Inflammatory reflex and baroreflex

The inflammatory reflex serves as a critical neuroimmune circuit, converting inflammation detection into reflexive inhibition of pro-inflammatory cytokine production, predominantly via vagal efferents through the cholinergic anti-inflammatory pathway (CAP; Pavlov and Tracey, 2012). This mechanism not only preserves immune homeostasis but also mitigates the risk of tissue damage from excessive inflammation (Caravaca et al., 2022a). Complementarily, the baroreflex, primarily recognized for cardiovascular regulation, adjusts heart rate and blood pressure in response to physiological stressors, thus influencing systemic inflammation. Recent studies underscore the therapeutic potential of these reflexes' synergy. Zhang et al. demonstrated that chronic VNS significantly improves autonomic control and attenuates heart failure development in a canine model, reflected by enhanced baroreflex sensitivity and reduced plasma levels of norepinephrine and angiotensin II, which are key mediators of cardiovascular and systemic inflammatory responses (Zhang et al., 2009). Simultaneous activation through VNS could enhance the management of inflammatory diseases with cardiovascular implications, such as atherosclerosis and myocardial infarction, broadening VNS's clinical applications. This integration of VNS in treating complex diseases showcases its dual therapeutic potential to modulate both inflammatory and cardiovascular dysfunctions.

### 2.2 Other neuro-immune mechanisms

#### 2.2.1 Vagus-adrenal axis

The vagus-adrenal axis is a crucial pathway through which VNS modulates systemic inflammation. Stimulation of the vagus nerve activates the hypothalamic-pituitary-adrenal (HPA) axis via vagal afferent fibers (Bonaz et al., 2019), leading to the release of corticotropin-releasing factor (CRF) from the hypothalamus. CRF then triggers the secretion of adrenocorticotropic hormone (ACTH) from the pituitary gland, which in turn stimulates the adrenal glands to produce cortisol and adrenaline. Cortisol downregulates pro-inflammatory pathways, such as nuclear factor κB NF-κB, and enhances anti-inflammatory proteins like annexin A1, while adrenaline further reduces inflammation by promoting immune cell resolution through beta-adrenergic receptors (Bonaz et al., 2017a). These hormonal responses collectively mitigate inflammation (Thrivikraman et al., 2013), positioning the vagus-adrenal axis as a key mechanism by which VNS exerts its therapeutic effects, particularly in hyperinflammatory conditions such as sepsis and systemic inflammatory response syndrome (SIRS).

#### 2.2.2 Neuropeptide-mediated pathways

Neuropeptides such as calcitonin gene-related peptide (CGRP) and vasoactive intestinal peptide (VIP) play pivotal roles in the anti-inflammatory effects of VNS. CGRP, secreted by sensory neurons, reduces pro-inflammatory cytokines and promotes vasodilation, aiding inflammation resolution (Fattori et al., 2021; Pavlov and Tracey, 2017). Conversely, VIP, by binding to immune cell receptors, diminishes the release of pro-inflammatory cytokines while boosting anti-inflammatory agents like IL-10 (Crosson and Talbot, 2023; Ganea et al., 2015). These interactions underscore the complexity and broad therapeutic potential of VNS in managing various inflammatory conditions.

### 2.3 VNS and metabolic regulation

#### 2.3.1 Vagus-Gut Axis: gateway to metabolic modulation

The Vagus-Gut Axis plays a pivotal role in metabolic regulation through VNS, influencing both gut motility and the microbial landscape. VNS enhances gastrointestinal motility and secretion, thereby improving digestion and nutrient absorption, which are crucial for maintaining metabolic homeostasis (Bonaz, 2022). This enhanced motility also facilitates a more favorable gut environment, potentially reducing the proliferation of pathogenic bacteria and supporting beneficial microbiota that contribute to anti-inflammatory states (Matteoli and Boeckxstaens, 2013). Furthermore, VNS upregulates the release of neurotransmitters like acetylcholine, which binds to α7 nicotinic acetylcholine receptors on intestinal epithelial cells, fortifying the gut barrier and mitigating the translocation of endotoxins into the systemic circulation (Bonaz et al., 2021). This barrier enhancement is vital for preventing systemic inflammation, a known contributor to metabolic syndromes such as diabetes and obesity. By modulating these neural and microbial pathways, VNS through the Vagus-Gut Axis presents a promising therapeutic avenue for treating metabolic diseases by leveraging the interconnected roles of neural signals and gut microbiota, thus providing a multi-faceted approach to disease management that extends beyond traditional pharmacotherapy.

#### 2.3.2 Vagus-Liver Axis: influencing glucose and lipid metabolism

The Vagus-Liver Axis plays a crucial role in the metabolic modulation exerted by VNS, specifically targeting key aspects of glucose and lipid metabolism that are central to metabolic syndrome pathogenesis. By activating the vagus nerve, VNS enhances glucose homeostasis through precise regulation of liver enzymes involved in gluconeogenesis and glycogenolysis, such as inhibiting phosphoenolpyruvate carboxykinase (PEPCK)—the rate-limiting enzyme in gluconeogenesis. This process, mediated through acetylcholine signaling via muscarinic receptors on hepatocytes, not only improves insulin sensitivity but also reduces hepatic glucose production, thereby offering a potent counter to hyperglycemia (Kawashima and Fujii, 2003; Pavlov and Tracey, 2012). Additionally, VNS influences lipid metabolism, potentially altering the expression of genes like SREBP-1c, a key regulator of fatty acid synthesis, thus affecting lipid transport and breakdown pathways. This neuromodulatory influence, while still under investigation for its direct mechanisms, suggests how VNS might mitigate dyslipidemia and lower cardiovascular disease risks associated with metabolic disturbances (Paton et al., 2013). Unlike traditional pharmacotherapies, which are often marred by side effects including gastrointestinal disturbances and cardiovascular risks, VNS provides a non-pharmacological, safer, and long-term therapeutic strategy for managing metabolic conditions like type 2 diabetes and hyperlipidemia, exemplifying a novel approach that leverages neural modulation to achieve metabolic balance with minimal adverse effects.

#### 2.3.3 VNS and mitochondrial function

VNS significantly influences mitochondrial function, a key factor in controlling inflammation and optimizing energy production (Nesci et al., 2023). VNS reduces reactive oxygen species (ROS) and stabilizes mitochondrial membrane potential, thereby mitigating oxidative stress, a primary contributor to chronic inflammation and cellular damage (Zhang et al., 2024). It also regulates mitochondrial dynamics by balancing fusion and fission processes, modulating proteins such as Drp1 and Mfn1/2, especially in myocardial ischemia models (Prathumsap et al., 2022). This regulation is supported by the activation of the Nrf2/HO-1 signaling pathway, which strengthens cellular antioxidant defenses and protects mitochondria from oxidative harm (Zhang et al., 2024). Furthermore, VNS activates the AMPK signaling pathway, specifically through the M3R/CaMKKβ/AMPK axis (Xue et al., 2017), enhancing mitochondrial biogenesis and energy metabolism. This metabolic boost is complemented by increased glucose uptake and ATP production via upregulation of Glut4 and CPT1α (Luo et al., 2020), underscoring VNS's capacity to enhance mitochondrial energy efficiency. These mechanisms collectively position VNS as a potential therapeutic approach for conditions characterized by mitochondrial dysfunction and chronic inflammation.

#### 2.3.4 Hormonal regulation through vagus nerve stimulation

VNS profoundly influences hormonal regulation, which plays a pivotal role in metabolic homeostasis and energy balance. By activating the vagus nerve, VNS can modulate the secretion of critical metabolic hormones, including insulin and glucagon, which are essential for glucose regulation. This modulation helps maintain glucose homeostasis, potentially benefiting conditions like diabetes by improving insulin sensitivity and reducing glycemic variability (Sorski and Gidron, 2023; Das, 2011). Moreover, VNS has been shown to affect the levels of ghrelin and leptin, hormones that regulate hunger and satiety. This regulation can help address obesity and its related metabolic complications by altering eating behaviors and energy expenditure (Obst et al., 2020). Additionally, VNS impacts the HPA axis, which plays a critical role in stress response and metabolic processes. By modulating this axis, VNS can mitigate the adverse effects of chronic stress, further contributing to metabolic regulation (Das, 2011). The hormonal adjustments induced by VNS illustrate its potential as a comprehensive treatment modality that extends beyond traditional pharmacological therapies, offering a holistic approach to managing metabolic and inflammatory diseases.

#### 2.3.5 Integration of neuro-metabolic pathways in VNS

VNS uniquely integrates neuro-metabolic pathways, expanding its therapeutic applications beyond traditional neuromodulation by merging anti-inflammatory responses with metabolic regulation. It adjusts neurotransmitter and hormone secretion, influencing functions like insulin release, glucose homeostasis, lipid metabolism, and energy expenditure. Notably, VNS activates the cholinergic anti-inflammatory pathway, reducing pro-inflammatory cytokines and enhancing insulin sensitivity and glucose uptake. It also affects the hypothalamic-pituitary-adrenal axis and gut hormones such as ghrelin and leptin, linking neural activity with metabolic outcomes and impacting appetite and energy regulation. This comprehensive modulation offers a novel therapeutic approach for conditions like type 2 diabetes, obesity, and metabolic syndrome. While VNS can cause side effects such as voice alteration, throat discomfort, and coughing, it offers advantages over pharmacotherapies by potentially reducing systemic side effects like hypoglycemia, gastrointestinal issues, and weight gain, and by targeting multiple pathways simultaneously. Future research should delineate the specific neural circuits and mechanisms to optimize VNS protocols, potentially revolutionizing treatment strategies for metabolic and inflammatory disorders.

### 2.4 Emerging mechanisms and interactions

#### 2.4.1 Microbiota-vagus interactions

The interaction between the gut microbiota and the vagus nerve is a dynamic area of research with substantial implications for the anti-inflammatory effects of VNS (Siopi et al., 2023; Bonaz et al., 2018). The gut microbiota maintains immune homeostasis, and its imbalance is associated with chronic inflammation in conditions like IBD and metabolic syndrome. VNS modifies the gut microbiota, promoting beneficial bacteria and reducing harmful ones (Han et al., 2022). This microbiota modulation by VNS introduces a novel therapeutic approach to reestablish immune equilibrium and decrease systemic inflammation, demonstrating the potential for microbiota-targeted VNS interventions in complex inflammatory disorders.

#### 2.4.2 Crosstalk with central nervous system

**Figure 1:**
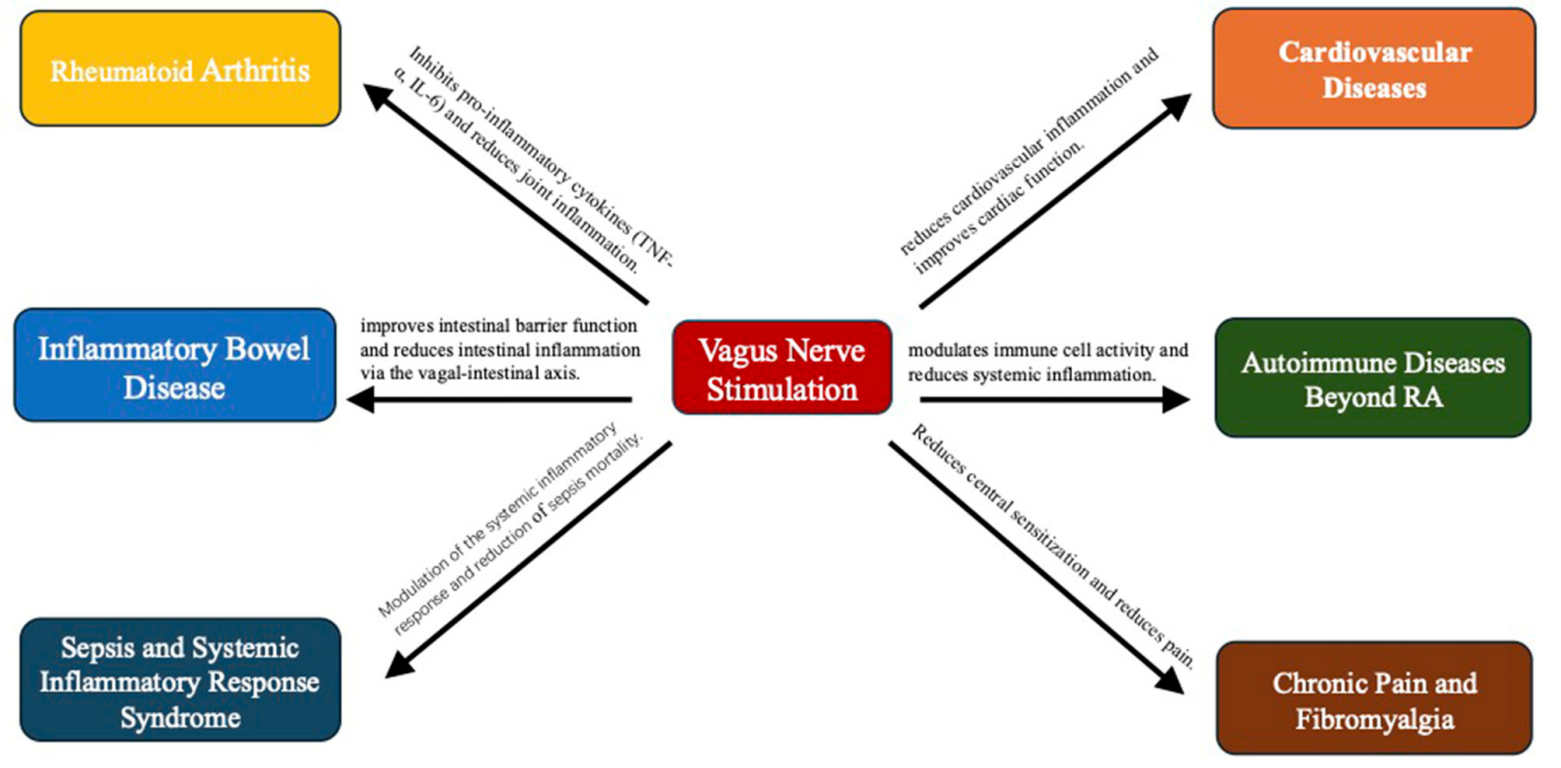
Vagus nerve stimulation in anti-inflammatory therapy: comprehensive mechanistic pathway.

The central nervous system (CNS) plays a dual role as both regulator and target of inflammation, with VNS exerting profound effects on neuroinflammatory pathways. VNS modulates neurotransmitter release and cytokine production, thereby reducing neuroinflammation and shielding neurons in conditions like multiple sclerosis, Parkinson's disease, and Alzheimer's disease. The interplay between VNS and the CNS illuminates its therapeutic potential for neuroinflammatory and neurodegenerative diseases. As insights into these interactions expand, VNS is poised to become integral in developing new management strategies for a broad spectrum of inflammatory conditions involving CNS dysfunction. A comprehensive overview of the mechanistic pathways by which vagus nerve stimulation exerts its anti-inflammatory effects is illustrated in Figure 1.

## 3 Expanded clinical applications of vagus nerve stimulation

### 3.1 Rheumatoid arthritis

Rheumatoid arthritis (RA) is a chronic autoimmune disorder characterized by persistent synovial inflammation and progressive joint destruction, with a hallmark of immune dysregulation that leads to excessive production of pro-inflammatory cytokines, driving ongoing tissue damage (Smolen et al., 2016). Despite the relief provided by disease-modifying antirheumatic drugs (DMARDs) and biologics for many patients, these treatments often entail severe side effects and do not fully control the disease in all patients (Araújo et al., 2016). In this context, non-invasive VNS (nVNS) has emerged as an innovative adjunct therapy, showing promise in effectively modulating immune responses. Clinical studies report significant reductions in RA symptoms and inflammatory biomarkers, such as C-reactive protein (CRP), with nVNS, alongside decreases in joint pain and swelling and improvements in patient-reported quality of life, which underscores its capacity to enhance daily functioning (Marsal et al., 2020a, 2021; Mondal et al., 2021).

Some patients even maintain improved symptoms post-treatment, suggesting nVNS's potential for inducing lasting anti-inflammatory effects. Notably, nVNS reduces key pro-inflammatory cytokines like TNF-α and interleukin-6 (IL-6), which corresponds with overall health improvements (Drewes et al., 2021). Additionally, nVNS complements traditional RA therapies by enhancing their effectiveness (Marsal et al., 2020b), reducing necessary dosages, and minimizing adverse effects, establishing itself as a safe, effective, and viable alternative to conventional immunosuppressive therapies and offering a more targeted approach to managing this complex disease.

### 3.2 Inflammatory bowel disease

Inflammatory bowel disease, which includes Crohn's disease and ulcerative colitis (Hodson, 2016), is marked by a chronic, unregulated immune response within the gastrointestinal tract. The introduction of nVNS marks a significant advancement in treating IBD by targeting the vagal-intestinal axis (Mikami et al., 2022; Breit et al., 2018). This method enhances cholinergic signaling, diminishes mucosal inflammation, bolsters intestinal barrier integrity, and suppresses key pro-inflammatory cytokines like TNF-α and IL-6 (Bonaz, 2024). Clinical research highlights that reductions in fecal calprotectin—a critical inflammation marker—align with better clinical remission rates and enhanced life quality, establishing nVNS as a potent supplement to existing therapies, especially in treatment-resistant cases.

Moreover, nVNS mitigates inflammatory responses, boosts intestinal motility, and lessens reliance on conventional drugs, thus reducing their side effects and associated risks (Cirillo et al., 2022; Yeshi et al., 2020). Recent trials further validate nVNS's role in alleviating common IBD symptoms such as abdominal pain, diarrhea, and intestinal bleeding, supported by a robust safety profile with minimal adverse effects. Significant reductions in inflammatory biomarkers continue to substantiate nVNS's anti-inflammatory properties (D'Haens et al., 2023; Bonaz et al., 2017b). However, larger randomized controlled trials are necessary to definitively ascertain nVNS's long-term efficacy and safety. Research is also exploring how to optimize stimulation settings like frequency and intensity to tailor treatment protocols (Bonaz and Sinniger, 2023). In summary, nVNS is emerging as an essential tool in IBD management, offering a novel, effective option for patients unresponsive to standard treatments and potentially reshaping the landscape of translational medicine as investigations advance.

### 3.3 Sepsis and systemic inflammatory response syndrome

Sepsis and Systemic Inflammatory Response Syndrome (SIRS) are critical conditions characterized by an overwhelming immune response, leading to widespread inflammation, organ dysfunction, and high mortality (Chakraborty and Burns, 2024; Bosmann and Ward, 2013). At the onset of SIRS, immune cells like macrophages and lymphocytes release numerous inflammatory mediators, such as TNF-α and interleukins, initiating both local and systemic responses. This hyperinflammatory state often results in microcirculatory disorders, damaging microvasculature, decreasing blood flow, and causing tissue hypoxia that exacerbates organ dysfunction (Jentzer et al., 2020). Additionally, SIRS can disrupt immune regulation, lowering resistance to infections and elevating the risk of secondary complications.

Despite ongoing advancements in sepsis management, truly effective treatment options are still scarce. In this landscape, nVNS has shown promise in preclinical and early clinical studies as a method to control systemic inflammation and enhance survival outcomes (Komegae et al., 2018). nVNS works by mitigating the cytokine storm associated with septic shock, reducing levels of inflammatory mediators such as TNF-α, IL-1β, and HMGB1. Preliminary trials indicate that nVNS may also stabilize hemodynamic parameters and lessen the need for vasopressors (Van Beekum et al., 2021), underscoring its potential as a supportive therapy in critical care settings. This evolving evidence positions nVNS as a groundbreaking tool in managing these complex, life-threatening conditions.

### 3.4 Cardiovascular diseases

Chronic inflammation is central to the development of cardiovascular diseases such as atherosclerosis, myocardial infarction, and heart failure (Fearon and Fearon, 2008). Inflammatory processes initiate the accumulation of immune cells within arterial walls, causing endothelial damage and arterial thickening, which promote atherosclerosis and increase cardiovascular risk (Sohrab et al., 2023; Distler et al., 2021). Post-myocardial infarction, inflammation intensifies cardiac remodeling and contributes to heart failure progression. VNS, especially in its nVNS, offers a potential therapeutic approach by activating the cholinergic anti-inflammatory pathway, reducing key inflammatory markers such as C-reactive protein (CRP) and IL-6.

Early animal studies underscore the benefits of chronic VNS in enhancing long-term survival, preventing cardiac pump failure, and mitigating remodeling (Alfaddagh et al., 2020). Recent focus has shifted to low-level VNS (LL-VNS) as a non-invasive technique to improve cardiovascular autonomic function and suppress inflammation (Sorriento and Iaccarino, 2019). Although VNS has shown potential in animal models for managing hypertension and enhancing cardiovascular outcomes, clinical trials like NECTAR-HF and INOVATE-HF have produced mixed results, highlighting the necessity for better patient selection and protocol optimization (Cucu, 2022; Anzai, 2018). As such, continuing research is vital to fully realize the therapeutic promise of VNS in cardiovascular disease management, confirming its efficacy and safety in hypertensive patients through detailed, rigorous randomized controlled trials (Gierthmuehlen et al., 2020; Li et al., 2024). This ongoing investigation will be crucial in integrating VNS into broader cardiovascular treatment frameworks.

### 3.5 Autoimmune diseases beyond RA

The therapeutic reach of nVNS extends beyond rheumatoid arthritis (RA), benefitting other autoimmune diseases such as systemic lupus erythematosus (SLE), multiple sclerosis (MS), and psoriasis (Ramkissoon et al., 2021; Tynan et al., 2022). In SLE, nVNS has demonstrated effectiveness in reducing autoantibody titers and mitigating disease flares by influencing B-cell activity and cytokine profiles, presenting a viable strategy for managing systemic inflammation (Bazoukis et al., 2023). Similarly, early evidence suggests that nVNS may alleviate neuroinflammation and decelerate disease progression in MS, attributes likely linked to its neuroprotective effects and ability to modulate immune responses (Marrosu et al., 2007).

In the case of psoriasis, nVNS has shown potential in clinical trials to lessen the severity of skin lesions by suppressing Th17-related cytokines, pivotal in driving inflammation in this condition (Genovese et al., 2020). These insights are supported by broader VNS mechanisms, including the activation of the cholinergic anti-inflammatory pathway and direct modulation of immune cells like macrophages and lymphocytes (Fang et al., 2023). The diverse applications of nVNS highlight its role as a safer and more targeted alternative to conventional immunosuppressive therapies, offering significant promise for enhancing patient outcomes across a spectrum of inflammatory autoimmune disorders. This breadth of applicability underscores nVNS's potential as a transformative tool in autoimmune disease management.

### 3.6 Chronic pain and fibromyalgia

Chronic pain and fibromyalgia, characterized by heightened central sensitization and dysregulated inflammatory responses (Bentley et al., 2018), often pose significant challenges to traditional pharmacological treatments, which frequently offer limited success and notable side effects (Martinez and Guimarães, 2024; Cohen et al., 2021). Non-invasive VNS presents a promising alternative, effectively targeting both peripheral and central inflammatory pathways. In fibromyalgia, significant efficacy has been observed with nVNS in reducing pain intensity, enhancing sleep quality, and improving overall wellbeing due to the suppression of pro-inflammatory cytokines and neuropeptides such as substance P and CGRP, along with normalization of autonomic dysfunction (Shao et al., 2023). Clinical studies support these outcomes, indicating substantial reductions in pain levels and improvements in symptoms associated with fibromyalgia, such as fatigue and anxiety (Johnson and Wilson, 2018; Demircioglu et al., 2024).

The therapeutic mechanisms of nVNS extend beyond the alleviation of symptoms, encompassing the reduction of systemic inflammation and the modulation of neurotransmitter release (Silberstein et al., 2020). These processes are of particular significance in the context of fibromyalgia pathology. Furthermore, the treatment maintains a favorable safety profile, with most side effects being mild and temporary, including throat discomfort and minor heart rate changes (Woodbury and Staats, 2024). The efficacy and safety of VNS in managing chronic pain have been confirmed by systematic review and meta-analysis, leading to its FDA approval for certain pain conditions (Toffa et al., 2020; Cai et al., 2024). However, further research is needed to optimize treatment parameters for individual patients and to explore nVNS's long-term effectiveness and safety in fibromyalgia and other chronic pain conditions (Ananda et al., 2023). Additionally, future investigations should consider the benefits of integrating nVNS with other therapeutic modalities, such as pharmacotherapy and psychotherapy, to enhance patient outcomes.

### 3.7 Asthma and chronic obstructive pulmonary disease

Asthma and chronic obstructive pulmonary disease (COPD) are chronic respiratory conditions marked by persistent airway inflammation and hyperresponsiveness (Abbaszadeh et al., 2022). While traditional treatments such as corticosteroids are effective, they often entail significant long-term side effects (Dey et al., 2022). nVNS has emerged as a promising adjunctive therapy, showing potential to mitigate both local and systemic inflammation (Bowles et al., 2022). In asthma, nVNS has been found to reduce eosinophil counts and lower levels of key cytokines such as IL-4 and IL-5, leading to improved respiratory outcomes (Mulders et al., 2015). Similarly, in COPD, nVNS has proven effective in reducing systemic inflammation, enhancing lung function, and decreasing exacerbation frequency (Hilz, 2022). These benefits are likely mediated by nVNS's ability to modulate the inflammatory response, enhance lung function, and restore autonomic balance.

Furthermore, nVNS holds promise for treating other respiratory diseases, such as acute respiratory distress syndrome (ARDS), where it can help alleviate hypoxemia and pulmonary edema by tempering excessive inflammatory responses (Kaniusas et al., 2020). Advances in selective VNS techniques have enabled more precise modulation of the vagus nerve, improving therapeutic efficacy while minimizing side effects (Mastitskaya et al., 2021), notably in conditions like sleep-disordered breathing (Toffa et al., 2020; Kim et al., 2022). Despite these promising developments, extensive clinical trials are essential to confirm nVNS's long-term effectiveness and safety across various respiratory conditions. Future research should aim to clarify the molecular mechanisms by which nVNS influences respiratory inflammation, conduct rigorous randomized controlled trials, and develop tailored treatment protocols to optimize outcomes for patients with chronic respiratory diseases.

## 4 Innovations and future directions in non-invasive VNS

### 4.1 Advances in device technology

The field of non-invasive VNS has experienced significant advancements in device technology, which have enhanced patient adherence and improved therapeutic outcomes. Traditional nVNS devices typically deliver stimulation through skin electrodes (Thompson et al., 2021) and have demonstrated efficacy in managing conditions like epilepsy and depression by reducing seizure frequency and improving mood states (Ryvlin et al., 2021). Recent technological developments have introduced devices equipped with real-time feedback mechanisms that monitor vital physiological markers such as heart rate variability and cytokine levels, allowing for the dynamic adjustment of stimulation parameters to optimize treatment efficacy (Goggins et al., 2022).

Additionally, ear-based VNS (taVNS) devices, which target specific areas within the ear, have been recognized for increasing patient compliance and have shown potential to boost cognitive functions and mood, especially in elderly or cognitively impaired individuals (Owens et al., 2024; Morris et al., 2019). Moreover, neck-based nVNS devices, focusing on cervical region stimulation, have the advantage of directly influencing multiple brain functions, although they may cause discomfort at the stimulation site (Toffa et al., 2020; Yap et al., 2020). The miniaturization and enhanced portability of these devices have broadened their use beyond traditional clinical environments, facilitating continuous or on-demand therapy that can be customized to meet individual patient needs. These technological innovations mark a substantial progression in the application of nVNS, establishing it as a more feasible and appealing option for the long-term management of chronic inflammatory conditions.

### 4.2 Precision neuromodulation

Precision neuromodulation stands at the cutting edge of neurostimulation technology, steering the progression of non-invasive VNS toward more tailored and efficacious therapies (Owolabi et al., 2023). This advanced approach enhances therapeutic outcomes by specifically targeting distinct portions of the vagus nerve (Bowles et al., 2022), achieving greater efficacy and minimizing side effects compared to traditional whole-nerve stimulation methods. Precision VNS operates by selectively stimulating different fiber bundles within the vagus nerve (Mourdoukoutas et al., 2018), enabling exact control over physiological pathways responsible for functions like heart rate regulation (Wernisch et al., 2024), without impacting unrelated systems such as digestion or immune responses (Ahmed et al., 2022).

Technological innovations have further refined this approach by incorporating real-time feedback mechanisms that adjust stimulation parameters—intensity and frequency—based on real-time physiological data from the patient (Austelle et al., 2022). This adaptation enhances treatment flexibility and effectiveness, allowing for a responsive and dynamic therapeutic process. The integration of various biomarkers, including cytokine levels, heart rate variability, and EEG patterns, into treatment protocols enables clinicians to continuously customize VNS therapy to meet the specific, changing needs of each patient (Sauer et al., 2024). As precision neuromodulation evolves, it promises to significantly shape the future of nVNS, positioning it as a pivotal element of personalized medicine and expanding its application across a broad spectrum of inflammatory and neurological disorders.

### 4.3 Combination therapies

The integration of non-invasive VNS with other therapeutic modalities offers a promising avenue to enhance efficacy and tackle treatment resistance in complex inflammatory conditions (Abdullahi et al., 2023; Yu and Wang, 2023). Combining nVNS with pharmacological treatments, such as anti-inflammatory drugs or biologics, has been shown to produce synergistic effects that amplify therapeutic outcomes. For example, nVNS could enhance the effects of TNF inhibitors in rheumatoid arthritis or reduce the necessary dosages of corticosteroids in asthma, thus minimizing drug-related side effects and improving overall patient outcomes (Sauer et al., 2024).

Additionally, nVNS can be effectively paired with behavioral interventions, like cognitive-behavioral therapy (CBT), to improve outcomes in conditions such as depression and anxiety (Hays et al., 2023). Research indicates that VNS can boost the therapeutic effects of behavioral therapies by modulating neurochemicals that facilitate neuroplasticity, thereby enhancing cognitive functions such as learning and memory (Engineer et al., 2017). Moreover, when combined with other neuromodulatory techniques like transcranial magnetic stimulation (TMS) or transcutaneous electrical nerve stimulation (TENS), nVNS may offer complementary benefits, further improving autonomic balance and effectively controlling chronic inflammation (Sackeim, 2021).

Despite the promising potential of these combination therapies, several challenges remain in their clinical implementation, including the necessity for tailored treatment plans, enhanced patient education, and robust regulatory support (Güvenç Paltun et al., 2021). However, exploring these multimodal strategies opens new pathways for maximizing the therapeutic potential of nVNS, offering a flexible and comprehensive approach to managing complex inflammatory and neurological conditions.

## 5 Challenges and considerations for clinical implementation

Despite the promising advancements and broad therapeutic potential of non-invasive VNS, integrating this therapy into mainstream clinical practice faces several challenges.

### 5.1 Regulatory and accessibility challenges

The clinical integration of non-invasive VNS faces substantial regulatory and accessibility challenges that hinder its widespread adoption. Regulatory approval processes for nVNS devices exhibit significant variability across different regions (Kaç et al., 2023; Forum on et al., 2015), where stringent regulatory demands can delay market access and restrict patient availability. This inconsistency is compounded by the absence of standardized guidelines for the evaluation of nVNS's efficacy and safety, which often extends approval timelines and escalates costs for manufacturers (Amon et al., 2024). Furthermore, the high initial investment required for nVNS devices, along with their ongoing maintenance and replacement costs (Morris et al., 2016), constitutes a considerable financial barrier, particularly in resource-constrained settings. The situation is exacerbated by inconsistent insurance coverage, which limits accessibility for the populations that stand to benefit the most from this non-invasive, potentially cost-effective therapy.

To navigate these obstacles, it is imperative to foster coordinated actions aimed at streamlining regulatory pathways, reducing costs through technological innovations and economies of scale, and advocating for more comprehensive insurance coverage. Such strategic measures are crucial for enhancing the accessibility of nVNS, rendering it a more feasible option across diverse healthcare environments, and potentially revolutionizing its role in clinical practice.

### 5.2 Patient compliance and education

Patient compliance is crucial for the success of nVNS therapy (Jin et al., 2008). Unlike pharmacological treatments, nVNS requires active patient participation, which typically involves daily or on-demand use of the device. Ensuring adherence to prescribed treatment regimens is vital for achieving optimal outcomes, yet it presents a significant challenge without proper education and support (Thummak et al., 2023). Educational programs are essential in providing patients with comprehensive knowledge about how nVNS works, its expected benefits, and the critical importance of consistent use. Healthcare providers play a central role in this educational effort by offering thorough device training, delivering clear usage instructions, and addressing patient concerns to improve adherence (Gold and McClung, 2006). Moreover, incorporating nVNS into comprehensive care plans that include regular follow-ups, monitoring, and feedback can significantly boost patient engagement and compliance. Advances in user-friendly device design, minimal maintenance requirements, and integration with mobile health technology are additional strategies that can facilitate easier use and enhance patient compliance.

### 5.3 Research gaps and future studies

While nVNS demonstrates significant promise in managing inflammatory conditions, there are several research gaps that need addressing to fully optimize its clinical application. Longitudinal studies are essential to determine the long-term efficacy and safety of chronic nVNS therapy (Ferstl et al., 2024; Lerman et al., 2019). Furthermore, a critical area of ongoing research is the optimization of stimulation parameters tailored to diverse patient populations (Thompson et al., 2021). Investigating biomarkers that can predict patient response to nVNS is pivotal for developing personalized treatment protocols (Ravan et al., 2017), which could significantly enhance both efficacy and safety. Additionally, exploring the potential of nVNS in novel applications, such as treating neurodegenerative diseases and cancer-related inflammation, could expand its therapeutic scope. Addressing these research gaps through comprehensive clinical trials and translational studies is fundamental to advancing the field of nVNS, paving the way for broader clinical adoption and enhanced patient outcomes.

## 6 Summary and outlook

This comprehensive review has synthesized the transformative potential of VNS in anti-inflammatory therapy, emphasizing its efficacy across a broad spectrum of inflammatory and autoimmune conditions as supported by robust clinical applications and pre-clinical models. VNS effectively mitigates symptoms in disorders such as rheumatoid arthritis, inflammatory bowel disease, and epilepsy, offering a viable alternative to pharmacological interventions by leveraging mechanisms such as the cholinergic anti-inflammatory pathway, vagus-adrenal axis, and vagus-gut axis. Technological advancements in precision neuromodulation and device design have not only enhanced therapeutic outcomes but also improved patient compliance, broadening VNS's appeal for long-term management of chronic inflammatory diseases.

However, the wider adoption of VNS faces challenges including regulatory complexities, high costs, and inconsistent insurance coverage, necessitating strategic approaches to overcome these barriers. Ensuring consistent and correct device use is critical for achieving optimal outcomes, with future research needing to focus on developing personalized protocols that enhance efficacy while minimizing side effects. By expanding VNS applications to include emerging conditions such as neurodegenerative diseases and integrating it into comprehensive treatment plans that include pharmacological and behavioral therapies, VNS can provide synergistic effects, enhancing anti-inflammatory benefits and patient quality of life. As the field progresses, VNS is poised to revolutionize the management of complex inflammatory disorders, promising a more effective and personalized therapeutic strategy that aligns with current scientific and clinical paradigms.
